# Study of the Inhibitory Effects of Enteral Nutrition Formula on Indomethacin-Induced Gastric Lesions in Mice

**DOI:** 10.3390/nu11123058

**Published:** 2019-12-14

**Authors:** Takeshi Yoshimi, Yoshiaki Yamagishi, Issei Kanegawa, Megumi Suda, Rei Saiki, Ken-ichiro Tanaka, Hitomi Goda, Toshiyuki Kudo, Kiyomi Ito

**Affiliations:** 1 Research Institute of Pharmaceutical Sciences, Musashino University, 1-1-20 Shinmachi, Nishitokyo-shi, Tokyo 202-8585, Japan; t.yoshimi68@gmail.com (T.Y.); y_yama@musashino-u.ac.jp (Y.Y.); s1343052@stu.musashino-u.ac.jp (I.K.); s1443120@stu.musashino-u.ac.jp (M.S.); s1543108@stu.musashino-u.ac.jp (R.S.); k-tana@musashino-u.ac.jp (K.-i.T.); h_goda@musashino-u.ac.jp (H.G.); to_kudou@musashino-u.ac.jp (T.K.); 2 Department of Pharmacy, Ogikubo Hospital, 3-1-24 Imagawa, Suginami-ku, Tokyo 167-0035, Japan

**Keywords:** non-steroidal anti-inflammatory drugs, enteral nutrition formula, gastric lesions

## Abstract

We investigated the effects of enteral nutrition formula on non-steroidal anti-inflammatory drug (NSAID)-induced gastric lesions in mice. Male ICR mice aged 7–9 weeks old were fasted, then orally given either purified water, Mermed^®^ One, or 2-fold diluted Terumeal^®^ 2.0α as enteral nutrition (25 or 50 mL/kg each). Indomethacin (IND) was orally administered at 20 mg/kg after 30 min, and the stomach was removed 6 h later and fixed in formalin. The number and area of lesions in the stomachs of the mice given enteral nutrition showed a significant, dose-dependent decrease compared to the purified water-treated group, and no significant difference was seen between the two enteral nutrition-treated groups. Comparable time courses of plasma IND concentrations suggest that enteral nutrition does not inhibit gastrointestinal absorption of IND. Our findings indicate that administering enteral nutrition could inhibit the onset of NSAID-induced gastric ulcers.

## 1. Introduction

Non-steroidal anti-inflammatory drugs (NSAIDs) are widely used in clinical situations to suppress inflammation in orthopedic diseases, to inhibit platelet aggregation in the treatment and prevention of coronary artery disease (with low-dose aspirin), and for many other purposes. NSAIDs work by inhibiting the prostaglandin (PG) synthase cyclooxygenase (COX) in the arachidonic acid cascade, thereby suppressing PG production in inflamed areas to provide anti-inflammatory action. These drugs, however, also cause NSAID-induced ulcers by inhibiting the gastric mucosal protective effects of PG [1,2], characteristically producing relatively shallow ulcers predominantly in the pyloric antrum [3]. Since recent studies show that NSAID-induced ulcer is caused by the simultaneous inhibition of both COX-1 and COX-2, efforts have been continued to develop new COX inhibitors with selectivity for either COX-1 or COX-2 [4,5]. Although such selective inhibitors are less likely to cause the undesirable gastrointestinal side effects, most of the currently available NSAIDs exhibit gastrointestinal mucosal damage as a common adverse reaction. Risk factors for NSAID-induced ulcers include a history of peptic ulcers, combination use of two or more NSAIDs, and advanced age (above 65–70 years old) [6,7]. Prophylaxis with a proton pump inhibitor (PPI) or PG analog is recommended for preventing ulcer recurrence in patients receiving combinations of NSAIDs and low-dose aspirin, or NSAID-induced bleeding ulcer in elderly patients [6,7].

Elderly patients are generally on more drugs than young patients [8], and therefore at greater risk of experiencing interactions between prophylactic drugs and other co-administered drugs [9]. Recent research has associated long-term oral PPI use with increased incidence of fundic gland polyps [10], increased risk of *Clostridium difficile* infections [11,12], and chronic diarrhea and collagenous colitis [13,14,15,16,17], suggesting that PPIs exert various negative effects on the digestive tract. Patients on PG drugs often experience diarrhea [6,7]. Limiting the number of prescription drugs a patient uses is therefore an effective strategy for mitigating these issues of drug interactions and adverse drug reactions among individuals receiving multiple drugs, as well as for reducing medical expenses.

Enteral nutrition formulas used for individuals unable to eat any or enough food are classified as drug formulas or food formulas. Some enteral nutrition food formulas contain sodium alginate, a fiber that causes them to thicken. Such enteral nutrition formulas are normally used to prevent aspiration pneumonia caused by reflux of the stomach contents, as the sodium alginate contained solidifies in the low-pH environment of the stomach [18], and studies have indicated that when applied in the clinic, these formulas could reduce gastrointestinal tract bleeding in patients with cholangitis caused by bile duct stones who have undergone endoscopic papillotomy or endoscopic biliary drainage [19]. No study, however, has shown enteral nutrition formulas to have any appreciable effect on gastric ulcers.

Many studies have pathologically evaluated the efficacy of substances such as sodium alginate [20], naturally occurring escin [21], and *Picrorhiza kurroa* rhizome extract [22] in gastric lesions induced in mice administered indomethacin (IND) orally. In order to investigate the possibility of enteral nutrition formula preventing gastric ulcer, we evaluated the effects of two enteral nutrition food formulas in this mouse model of IND-induced gastric lesions. The first, Mermed^®^ One, is a variable viscosity type liquid diet containing sodium alginate. The other, Terumeal^®^ 2.0α, has ingredients similar to the first agent but does not contain sodium alginate.

## 2. Materials and Methods

### 2.1. Reagents

The enteral nutrition formulas used were Mermed^®^ One (400 kcal/400 mL) and Terumeal^®^ 2.0α (400 kcal/200 mL). Each was purchased from Terumo Corporation (Tokyo, Japan) and their ingredients are listed in the supplementary table (Table S1). IND was purchased from Nacalai Tesque (Kyoto, Japan). Carboxymethyl cellulose (CMC), isoflurane, and 10% neutral buffered formalin were purchased from FUJIFILM Wako Pure Chemical Corporation (Tokyo, Japan). IND-d_4_ was purchased from Cayman Chemical (Ann Arbor, MI, USA). All other reagents used were of the highest commercially available grade.

### 2.2. Animals

Male ICR mice aged 7–9 weeks old (Sankyo Labo Service Corporation, Tokyo, Japan) were used. The temperature (23.3 ± 0.1 °C) and humidity (54.8 ± 1.1%) were maintained in an air-conditioned room and animals were housed in suitable cages with a 12-h light–dark cycle (lights on from 08:00 to 20:00 h) with free access to food and water unless otherwise stated. All animal experiments were conducted with the approval of the Institutional Animal Care and Use Committee of Musashino University.

### 2.3. Dosing and Blood Sampling Schedule

**Figure 1:**
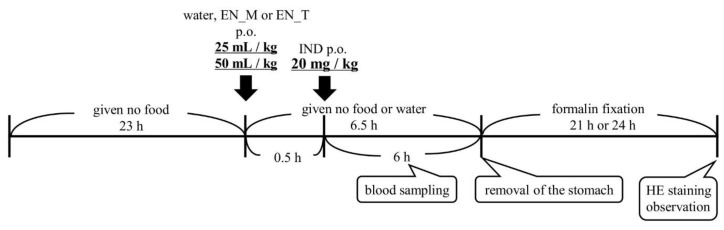
Experimental protocol. IND: indomethacin, EN_M: Mermed^®^ One, EN_T: Terumeal^®^ 2.0α diluted by a factor of 2 with purified water to reduce the caloric content to that of EN_M, HE: hematoxylin and eosin, p.o.: per os.

Mice were fasted for 23 h, administered either purified water or an enteral nutrition formula (25 or 50 mL/kg, orally), and then given IND (20 mg/kg, orally) 30 min later (*n* = 5 per group). The animals were given no food or water through to removal of the stomach under isoflurane anesthesia 6 h later. Mermed^®^ One (EN_M) and Terumeal^®^ 2.0α diluted by a factor of 2 with purified water to reduce the caloric content to that of EN_M (EN_T) were used as the enteral nutrition formulas. IND was administered as a 1% CMC suspension (2 mg/mL) (Figure 1).

An aliquot of 25–30 μL of blood was collected from the tail vein in a heparinized capillary tube (Hirschmann, Eberstadt, Germany) 0.25, 0.5, 1, 2, 4, and 6 h after IND administration. Just after collection, blood was centrifuged for 5 min at 14,800× *g* (H-1200F, Kokusan, Saitama, Japan), and the resulting plasma was stored at −80 °C. This part of the study was conducted on mice that received 25 mL/kg of purified water or enteral nutrition formula before IND administration (three groups).

### 2.4. Gross Observation of Gastric Mucosa and Histological Evaluation with Hematoxylin and Eosin Staining

The pyloric region of each removed stomach was ligated, and the stomach was filled by injecting 2 mL of 10% neutral-buffered formalin into the cardiac region followed by either observation using magnifying glass (*n* = 3) or histological evaluation (*n* = 2) as follows (Figure 1):

#### 2.4.1. Observation of Gastric Lesions Induced by IND

The mucosal surface was fixed for 21 h. Following fixation, an incision was made along the greater curvature to open the stomach. A magnifying glass with ×7 magnification (Peak Scale Lupe 7×, Tohkai Sangyo, Tokyo, Japan) was used to determine the number and area (in square millimeters) of gastric lesion sites (four groups for each dose).

#### 2.4.2. Histological Evaluation

After fixation for about 24 h, each stomach was opened with an incision along the greater curvature. Tissue from the left side of the pyloric portion was removed and embedded in paraffin to create tissue blocks. Embedding was contracted to an external facility (New Histo. Science Laboratory, Tokyo, Japan). Tissue blocks were sectioned using a Leica RM2245 microtome (Leica Biosystems, Wetzlar, Germany). The resulting 4-μm sections were placed on slides and stained with hematoxylin and eosin (HE) using standard techniques. This part of the study was conducted on both untreated mice and mice that received 25 mL/kg of purified water or enteral nutrition formula before IND administration (four groups).

### 2.5. Analysis of Plasma IND Concentrations

90 μL of acetonitrile (containing 2.5 μM IND-d_4_ as the internal standard) was added to 10 μL of each plasma sample, and the resulting mixture was vortexed for 30 s. Samples were then centrifuged (4 °C, 8000× *g*, 10 min) using an Eppendorf 5427R centrifuge (Eppendorf, Hamburg, Germany). The IND concentration in the supernatant was determined with a liquid chromatography tandem-mass spectrometry (LC-MS/MS) instrument (LCMS-8040; Shimadzu, Tokyo, Japan). The column was a GL sciences Inertsil ODS-3 (particle size: 5 μm; inside diameter: 4.6 mm; length: 150 mm, GL sciences, Tokyo, Japan), the mobile phase was a 3:7 mixture of purified water (0.1% formic acid) and acetonitrile (0.1% formic acid), the column temperature was 40 °C, the flow rate was 0.5 mL/min, and ionization conditions were electrospray ionization-positive, with *m/z* 358.00 > 139.05 IND and *m/z* 362.10 > 142.85 IND-d_4_ (internal standard) detected as ions.

### 2.6. Calculation of Pharmacokinetic Parameters 

The trapezoidal rule was used to calculate the area under the plasma concentration-time curve to 6 h postdose (AUC_0–6_) from time courses of plasma IND concentrations. Maximum plasma concentration (C_max_) and time to C_max_ (T_max_) were determined from time courses of plasma IND concentrations. Mean residence time (MRT) was calculated by dividing the area under the first moment curve to 6 h postdose (AUMC_0–6_) as determined with the trapezoidal rule by AUC_0–6_.

### 2.7. Statistical Analysis

All data are expressed as mean ± standard deviation (SD).

Tukey’s test was performed using SPSS software (version 24; IBM, Tokyo, Japan) to test for significant differences. Values less than the critical rate of 5% (*p* < 0.05) were considered statistically significant.

## 3. Results

### 3.1. Effects of Enteral Nutrition Formula on IND-Induced Lesions

**Figure 2:**
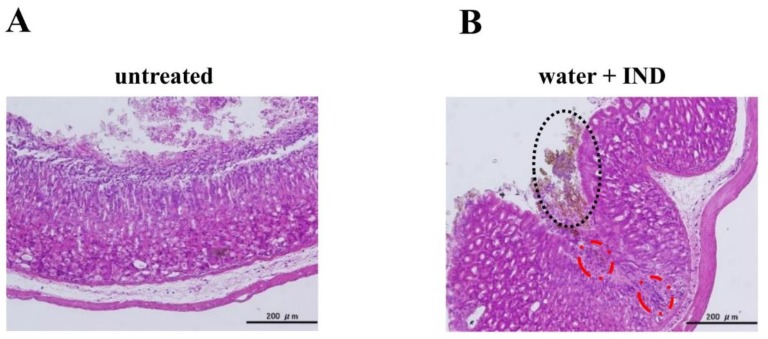
Histological evaluation of gastric mucosal lesions induced by IND. The stomach was removed, fixed in 10% neutral-buffered formalin for 24 h, and embedded in paraffin. Resulting tissue blocks were sectioned with a microtome and stained with HE stain. (**A**) Untreated mouse. (**B**) Mouse orally given purified water (25 mL/kg) 30 min before oral IND (20 mg/kg) administration, with the stomach removed 6 h later.

HE staining revealed no lesion formation in the tissues of the untreated group (Figure 2A), but showed markers of previous bleeding staining black and lesion formation in the group orally given purified water 30 min before oral IND (20 mg/kg) administration (water + IND group) (Figure 2B). Inflammatory cell infiltration was noted in the mucosa of sites where marks of previous bleeding were seen (Figure 2B).

**Figure 3:**
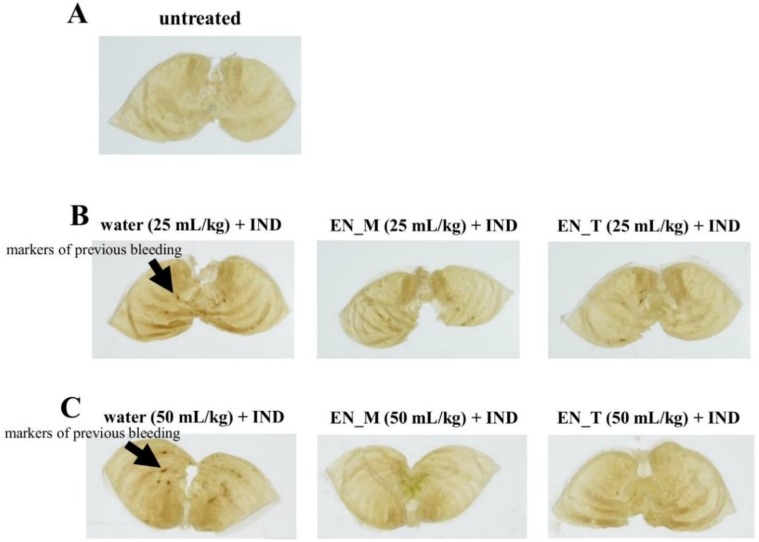
Effects of EN_M and EN_T on IND-induced gastric lesions. Figures show representative photographs of stomachs taken after fixation for 21 h in 10% neutral-buffered formalin following removal 6 h after oral IND (20 mg/kg) administration. (**A**) Untreated group. (**B**) Group treated with 25 mL/kg of purified water, EN_M, or EN_T 30 min before IND administration. (**C**) Group treated with 50 mL/kg of purified water, EN_M, or EN_T 30 min before IND administration.

Observations of formalin-fixed stomachs 6 h after oral IND administration revealed more markers of previous bleeding in the water + IND group than in the untreated group (Figure 3A–C). In contrast, few markers of previous bleeding were noted in the groups orally given 25 or 50 mL/kg of EN_M or EN_T 30 min before oral IND administration (EN_M + IND group and EN_T + IND group, respectively) (Figure 3B,C). Stomachs of the animals that received 50 mL/kg of EN_M or EN_T tended to have fewer markers of previous bleeding than those of animals receiving 25 mL/kg (Figure 3B,C).

**Figure 4:**
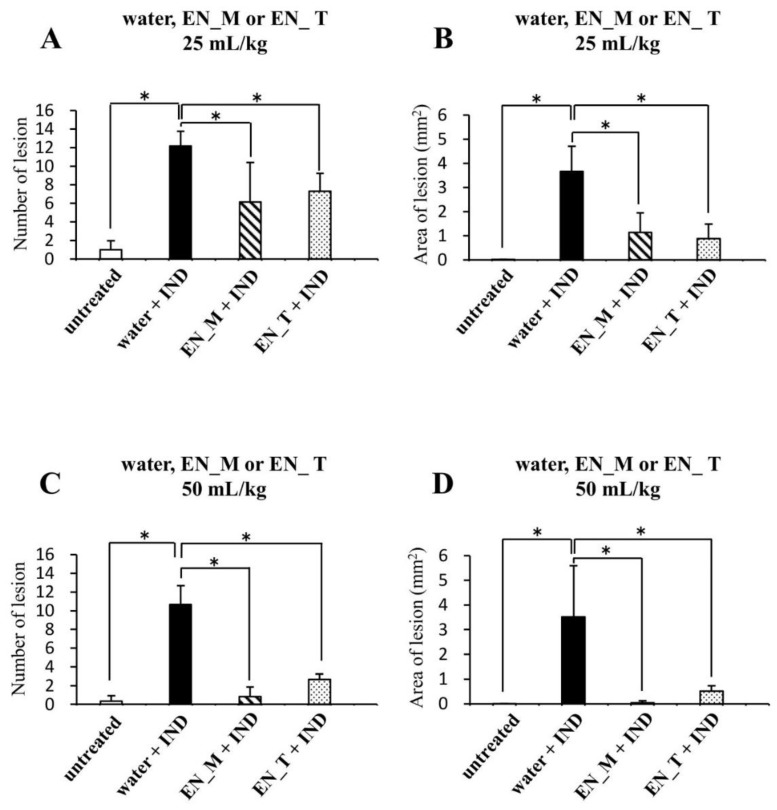
Effects of EN_M and EN_T on numbers and areas of gastric lesions induced by IND. Gross observations were performed to determine numbers (**A**,**C**), and areas (**B**,**D**), of lesions in stomachs taken after fixation for 21 h in 10% neutral-buffered formalin following removal 6 h after oral IND (20 mg/kg) administration. (**A**,**B**) Group treated with 25 mL/kg of purified water, EN_M, or EN_T 30 min before IND administration. (**C**,**D**) Group treated with 50 mL/kg of purified water, EN_M, or EN_T 30 min before IND administration. The open column represents the untreated group; the closed column, hatched column, and dotted column represents the group treated with purified water, EN_M, or EN_T, respectively, 30 min before IND administration. Each column and vertical bar represents mean + SD, *n* = 3. * *p* < 0.05 (Tukey’s test).

Determination of the number and area (in square millimeters) of gastric lesion sites using a magnifying glass revealed significantly more and larger lesions in the water + IND group than in the untreated group (Figure 4A–D). In contrast, numbers and areas of gastric lesion sites in the 25 mL/kg EN_M + IND and EN_T + IND groups were significantly smaller than in the water + IND group (Figure 4A,B). Moreover, numbers and areas of gastric lesion sites in the 50 mL/kg EN_M + IND and EN_T + IND groups were smaller than in the 25 mL/kg groups, showing that the anti-lesion effects of these enteral nutrition formulas were dose-dependent (Figure 4A–D). No significant differences in either parameter, however, were seen between EN_M + IND and EN_T + IND groups in any dosage level (Figure 4A–D).

### 3.2. Time Courses of Plasma IND Concentrations

**Figure 5:**
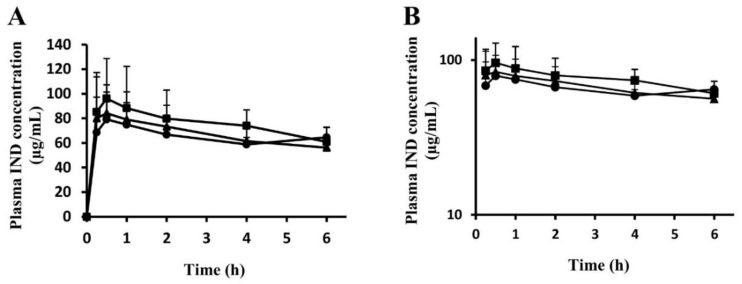
Effects of EN_M and EN_T on plasma IND concentrations. Blood was collected from the mice orally given 25 mL/kg of purified water, EN_M, or EN_T 30 min before oral IND (20 mg/kg) administration. LC-MS/MS was used to determine plasma IND concentrations. (**A**) Normal plot. (**B**) Semilog plot. The closed circles, triangles, and squares represent the profiles for the group treated with purified water, EN_M, or EN_T, respectively, 30 min before IND administration. Each symbol represents mean + SD, *n* = 5.

A comparison of time courses of plasma IND concentrations in the water + IND group and EN_M + IND and EN_T + IND groups revealed no significant inter-group difference at any time point (Figure 5A,B). Pharmacokinetic parameters were calculated from time courses. No significant inter-group differences were noted in AUC_0–6_, C_max_, T_max_, or MRT (Table 1).

## 4. Discussion

Our study began with an attempt to create a mouse model of IND-induced gastric lesions. No lesions were seen in the stomachs of the untreated group (Figure 2A and Figure 3A). Many lesions were noted in the stomachs of the water + IND group (Figure 2B and Figure 3B,C), and lesions in the water + IND group were more numerous and had a larger area than those in the untreated group (Figure 4). As in previous investigations [20,21,22], this attempt to create an animal model of gastric lesions with oral IND administration appears to have been successful.

To evaluate NSAIDs lesion-preventing effects of enteral nutrition formula, we orally administered two enteral nutrition formulas 30 min before orally administering IND. Observations of the gastric mucosal tissues and evaluation of lesion numbers and areas indicate EN_M and EN_T prevented the occurrence of IND-induced gastric lesions (Figure 2, Figure 3 and Figure 4). The preventative effect on lesion formation, moreover, appeared to increase with increasing doses of EN_M and EN_T.

Given that time courses of plasma IND concentrations (Figure 5A,B) indicate no significant inter-group differences in AUC_0–6_, C_max_, T_max_, or MRT (Table 1), EN_M and EN_T appear not to have affected gastrointestinal absorption of IND in the study. This finding rules out the possibility that EN_M and EN_T prevented gastric lesions by inhibiting IND absorption such that blood levels of IND were insufficient to cause gastric lesion formation.

NSAIDs-induced gastric lesions are currently thought to involve suppressed PG production due to cyclooxygenase inhibition and apoptosis triggered by mitochondrial dysfunction from NSAIDs deionized in the gastric fluid and taken into cells of the gastric mucosal epithelium [3,4]. Some ingredients in EN_M and EN_T may prevent gastric lesions by blocking one or both of these mechanisms underlying lesion formation. Common ingredients in both enteral nutrition formulas such as proteins, lipids, vitamins and minerals, rather than sodium alginate, seem to have contributed to this anti-lesion effect, because this effect was observed regardless of whether sodium alginate was present.

The Medication Pass Nutrition Supplement Program (Med Pass) is attracting attention as a way to prevent malnutrition in elderly people [23]. Under Med Pass, patients that must take multiple drugs receive them with enteral nutrition formula rather than water, and are thus able to nourish themselves and take their drugs simultaneously, even if they have difficulty in chewing. Consuming large amounts of water while taking multiple drugs can cause bloating, which can in turn reduce the amount of food consumed, thereby limiting nutrition intake from dietary sources. Elderly individuals are at particular risk of sarcopenia from weight loss caused by malnutrition, so this problem can initiate the frailty cycle [24]. Med Pass, a potentially effective solution to this problem, is drawing attention as a beneficial way to maintain quality of life in elderly individuals with this problem. Gastric ulcers are not the only adverse drug reactions associated with NSAIDs, which can also cause reduced appetite. This condition, when compounded with the use of multiple drugs, may promote malnutrition [25]. Our findings indicate that creating a Med Pass-like program that provides EN_M or EN_T to take with NSAIDs could help prevent NSAIDs-induced gastric ulcers and prevent malnutrition from the use of multiple drugs. This Med Pass suggestion could help alleviate malnutrition, but should be implemented with caution because the nutrients could cause drug interactions [26,27,28,29].

The demonstration of prophylactic effects of EN_M and EN_T on gastric ulcers in the study is significant from a clinical perspective. Showing that enteral nutrition, as a food formula, could alleviate gastrointestinal damage would help patients reduce the number of drugs they take by serving as an alternative to PPIs. Enteral nutrition formula could potentially reduce the risk of drug interactions and adverse reactions and reduce the number of prescription drugs patients must take, thereby helping to reduce soaring medical expenses.

## Figures and Tables

**Table 1:** Pharmacokinetic parameters calculated from time courses of plasma IND concentrations.

	Water + IND	EN_M + IND	EN_T + IND
T_max_ (h)	0.85 ± 0.70	0.50 ± 0.31	1.7 ± 2.4
C_max_ (µg/mL)	76.0 ± 35.6	88.8 ± 26.8	102 ± 22
AUC_0–6_ (µg h/mL)	385 ± 51	399 ± 82	435 ± 96
MRT	2.96 ± 0.21	2.84 ± 0.17	2.89 ± 0.35

Each value represents mean ± SD, *n* = 5. T_max_: time to C_max_, C_max_: maximum plasma concentration, AUC_0–6_: area under the plasma concentration-time curve to 6 h postdose, MRT: mean residence time.

## Supplementary Materials

The following are available online at https://www.mdpi.com/2072-6643/11/12/3058/s1, Table S1: Composition of the enteral nutrition formulas.
